# The relevance of magnetic resonance imaging in the era of Alzheimer's disease biomarkers

**DOI:** 10.1055/s-0045-1801863

**Published:** 2025-02-17

**Authors:** Marcio Luiz Figueredo Balthazar

**Affiliations:** 1 Universidade Estadual de Campinas, Faculdade de Ciências Médicas, Departamento de Neurologia, Campinas SP, Brazil.

Brain magnetic resonance imaging (MRI) has revolutionized how we view neurological diseases, with a level of anatomical detail that has yet to be surpassed by any other method. In several neurodegenerative diseases, and specifically in Alzheimer's disease (AD), instead of acting to rule out other potential causes, such as cerebrovascular, tumors, or inflammatory diseases, MRI has come to play an essential role in identifying patterns of atrophy. Different visual scales of atrophy have been incorporated into clinical practice, and, in AD, scales of mesial temporal atrophy such as that proposed by Scheltens 1 and of atrophy of the entorhinal cortex and transentorhinal region (ERICA), proposed by Enkirch, have been frequently used, with reasonable accuracy, as tools to support clinical diagnostic decisions. 2

However, the evolution of both research and diagnostic criteria has highlighted the importance of a more accurate diagnosis regarding the pathophysiology of the disease since atrophy is the final common pathway of different neurodegenerative processes and, therefore, is not very specific. Thus, complementary tests that quantify β-amyloid peptide, neuritic plaques, and tau protein have been validated, such as amyloid positron emission tomography (PET), tau PET, the dosage of these proteins in the cerebrospinal fluid and, more recently and still in the validation process, in the plasma. Thus, in the latest diagnostic criteria of the Alzheimer's Association of 2018 and 2024, the MRI evaluation was considered nonspecific for the diagnosis. 3 4

In the paper “Diagnosing preclinical and clinical Alzheimer's disease with visual atrophy scales in clinician practice”, Socher et al. retrospectively evaluated the specificity, sensitivity, accuracy, and positive and negative predictive values of the medial temporal atrophy (MTA) and ERICA scales according to the amyloid PET (11-C PiB) result and clinical diagnosis (cognitively normal, mild cognitive impairment, and Alzheimer's disease dementia). 5 They found that the sensitivity of the ERICA score in the AD group according to amyloid PET status was 77.7%, with an accuracy of 67.7%. MTA sensitivity was 40.7%, and the score specificity was 50% for clinical diagnosis. In mild cognitive impairment (MCI), both the ERICA and MTA scores achieved similar performances with an accuracy of ∼50%, with no differences in the visual analysis of cerebral atrophy. The ERICA score achieved moderate sensitivity at 70%, and the MTA score was more specific at 74%. 5

Considering the group with normal cognition, both ERICA and MTA scores achieved low sensitivities in this study, but the MTA score had a greater specificity (91%) than the ERICA score (60%). The authors conclude that clinicians could use this visual analysis, mainly the ERICA score, to screen for AD in patients with clinical dementia but not in asymptomatic or early stages without dementia. 5

Although the sensitivity, specificity, and accuracy values of the scales in this study were relatively low, their use may still be essential in clinical practice. It is common to find changes in these scales, even in people with initial symptoms. Furthermore, it is possible to quantify the volume of these structures more precisely using software (usually paid) available on the internet that defines, for example, the percentile in which a patient is concerning the population, considering age and sex.

Therefore, brain MRI continues to be an indispensable instrument in the evaluation of patients with cognitive problems, mainly due to the high cost and low availability of methods that directly assess the pathophysiology of AD.
